# Pollen parent affects rutin content of seeds of buckwheat (*Fagopyrum esculentum*)

**DOI:** 10.1270/jsbbs.24085

**Published:** 2025-06-18

**Authors:** Shiori Otsuka, Takashi Hara, Koji Ishiguro, Kenichi Matsushima, Yasuo Yasui, Katsuhiro Matsui

**Affiliations:** 1 Hokkaido Agricultural Research Center, National Agriculture and Food Research Organization (NARO), 9-4 Shinsei-Minami, Memuro, Kasai, Hokkaido 082-0081, Japan; 2 Graduate School of Life and Environmental Science, University of Tsukuba, 2-1-2 Kannondai, Tsukuba, Ibaraki 305-8602, Japan; 3 Institute of Agriculture, Academic Assembly Faculty, Shinshu University, 8304 Minamiminowa, Nagano 399-4598, Japan; 4 Graduate School of Agriculture, Kyoto University, Kitashirakawa Oiwake-cho, Sakyo-ku, Kyoto 606-8501, Japan; 5 Research Center of Genetic Resources, NARO, 2-1-2 Kannondai, Tsukuba, Ibaraki 305-8602, Japan

**Keywords:** buckwheat, rutin, pollen, xenia, RNA-seq, self-incompatibility

## Abstract

Buckwheat (*Fagopyrum esculentum*) is a heterostylous self-incompatible crop that requires outcrossing for seed formation. Pollen parents influence the traits of seeds in many plants, but their influence in buckwheat is unknown. Here, we crossed self-incompatible (SI) and self-compatible (SC) lines with different rutin contents. The average rutin contents of SI leading cultivars were all 0.15–0.21 mg/g, and a SI high rutin content line that we had developed was 0.6 mg/g, although it has wide range SDs (0.12–0.41). On the other hand, the average rutin contents of SC lines were 0.01–0.06 mg/g, with stable SDs (0.02–0.03). In crosses between high- and low-rutin parents, the average rutin content of F_1_ seeds was significantly lower than that of the high-rutin parent and higher than that of the low-rutin parent, indicating that the pollen parent influences the rutin content in seeds of buckwheat. RNA-seq analysis confirmed that alleles of several genes encoding enzymes involved in rutin synthesis derived from pollen parents were expressed during seed formation.

## Introduction

Buckwheat is a pseudocereal crop in the Polygonaceae and is widely grown, notably in Russia, China, France, and Japan (FAOSTAT 2023). It is used to make bread, noodles, and ethnic foods in combination with wheat, rice, or maize in many countries (Krkošková and Mrázová 2005). In Japan, buckwheat noodles have been eaten for more than 400 years and are considered a traditional food (Krkošková and Mrázová 2005, Qu *et al.* 2013). Known for their health benefits (Krkošková and Mrázová 2005), buckwheat grains contain abundant starch, vitamins, minerals, an well-balanced amino acid composition, fiber (Huda *et al.* 2021), rutin (Matsui and Walker 2020), a flavonoid with antioxidant, anti-inflammatory, anti-diabetic, anti-cancer, and pro-lipid-metabolism effects (Bhatt *et al.* 2022, Chu *et al.* 2014, Lee *et al.* 2016, Qu *et al.* 2013).

Since rutin is not present in other major crops, new buckwheat lines with a high rutin content are desired. However, it is difficult to develop new lines, because buckwheat is an outcrossing plant on account of heterostylous self-incompatibility (Matsui and Yasui 2020). Buckwheat has two flower types, pin and thrum; pin flowers have a long style and short stamens, whereas thrum flowers have a short style and long stamens (Darwin 1897). It is possible to cross only between plants with different flower types, so all resultant seeds are F_1_s with high heterozygosity (Matsui and Yasui 2020).

Self-compatible buckwheat lines have been developed from an interspecific cross between *Fagopyrum esculentum* and *Fagopyrum homotropicum* (Aii *et al.* 1998, Campbell 1995, Matsui *et al.* 2003, Wang *et al.* 2005, Woo *et al.* 1999). We developed the self-compatible line ‘Kyushu PL4’ (Matsui *et al.* 2008), which has been used as a maternal line to introduce self-compatibility into other lines, such as ‘Kyukei SC7’ (Hara *et al.* 2020, Takeshima *et al.* 2021, 2022). A PL4 genome database recently developed by a research group including ourselves (Fawcett *et al.* 2023) has provided much genetic information.

**Fig. 1.**
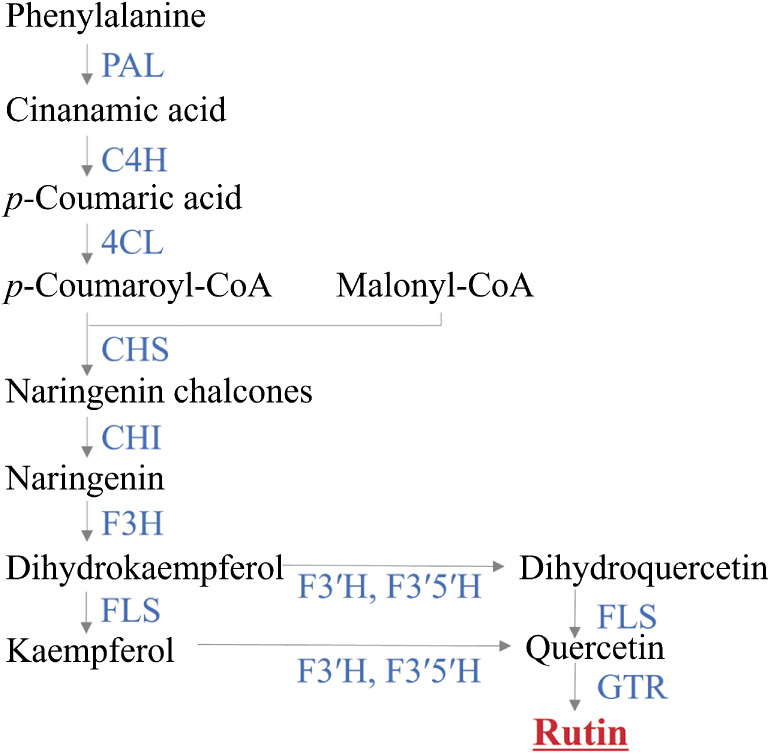
Rutin biosynthesis pathway in buckwheat. Enzymes are indicated in upper-case blue letters. Arrows indicate reactions catalyzed by the indicated enzymes. PAL, phenylalanine ammonia-lyase; C4H, cinnamate 4-hydroxylase; 4CL, 4-coumarate:Coa ligase; CHS, chalcone synthase; CHI, chalcone isomerase; F3H, flavanone 3-hydroxylase; F3ʹH, flavonoid 3ʹ-hydroxylase; F3ʹ5ʹH, flavonoid 3ʹ5ʹ-hydroxylase; FLS, flavonol synthase; GTR, flavonol glycosyltransferase.

Flavonoids, including rutin, also known as quercetin-glycoside-rhamnoside, are synthesized via the flavonoid biosynthesis pathway in several sequential steps within the phenylpropanoid biosynthesis pathway (Matsui and Walker 2020). Phenylalanine ammonia-lyase (PAL), cinnamate 4-hydroxylase (C4H), and 4-coumarate:CoA ligase (4CL) convert phenylalanine into *p*-coumaroyl-CoA. Chalcone synthase (CHS), chalcone isomerase (CHI), and flavone 3-hydroxylase (F3H) catalyze *p*-coumaroyl-CoA into dihydrokaempferol. From dihydrokaempferol, flavonoid 3ʹ-hydroxylase (F3ʹH), flavonoid 3ʹ5ʹ-hydroxylase (F3ʹ5ʹH), and flavonol synthase (FLS) produce quercetin (Matsui and Walker 2020, Zhang *et al.* 2017). The quercetin is then glycosylated by glycosyltransferases (GTRs) including glucosyltransferase (GT) and rhamnosyltransferase (RT) to produce rutin (Matsui and Walker 2020, Zhang *et al.* 2017) (Fig. 1). Although rutin is synthesized from quercetin, buckwheat seeds do not contain quercetin or contains quercetin only in trace amounts (Rauf *et al.* 2020, Sedej *et al.* 2012).

It is well known that pollen can influence the character of seeds or fruits, a phenomenon called xenia (effect on endosperm and embryos) or metaxenia (effect on surrounding tissues) (Denney 1992). For example, the pollen parent affects the fruit set, size, and mass of grapes and peonies (Sabir 2015, Xie *et al.* 2017); the mass and ripeness of highbush blueberries (Doi *et al.* 2021); the color of the seed coat of *Trifolium alexandrinum* (Malaviya *et al.* 2019); and the contents of chemical components in peonies, almonds, rapeseed, and *Siraitia grosvenorii* (Kodad *et al.* 2009, Sánchez-Pérez *et al.* 2012, Wang *et al.* 2010, Xie *et al.* 2017, Yan *et al.* 2019). As for flavonoids, the pollen parent affects the flavonoids content in figs and kiwiberries (Pourghayoumi *et al.* 2012, Stasiak *et al.* 2019).

Although buckwheat requires cross-pollination, little is known about the influence of pollen parents. Here, by measuring the rutin contents of F_1_ seeds produced by crosses between lines with high and low rutin contents, we clarified that the pollen parent influences the rutin content of F_1_ seeds. RNA-seq analysis of maturing F_1_ seeds detected several alleles related to rutin synthesis derived from parental lines.

## Materials and Methods

### Plant materials

We used five self-incompatible (SI) lines and two self-compatible (SC) lines (Supplemental Table 1). Four of the SI lines—‘Kitamitsuki’ (KTM), ‘Kitawasesoba’ (KTW), ‘Reranokaori’ (RRN), and ‘Kitayuki’ (KTY)—are leading cultivars in Hokkaido, Japan (Ohsawa 2020). By recurrent repeated individual selection for rutin content over 12 generations, we bred the SI ‘High Rutin content line No. 8’ (HR8) from ‘Botansoba’, which has high heterogeneity. We bred the SC line ‘Kyushu PL4’ (PL4) from a cross between *F. esculentum* and *F. homotropicum* (Matsui *et al.* 2008, Matsui and Yasui 2020). We also selected a low-rutin-content SC line (LoR) from an F_4_ segregating line produced from a cross between SC ‘Kyukei SC2’ (Matsui *et al.* 2003) and SC ‘C0408-0RP’, which was bred at Kade Research Ltd. (Hara *et al.* 2011).

### Production of parental line and F_1_ seeds in a field for measurement of rutin content

The parental line listed in Supplemental Table 1 and F_1_ seeds produced by the cross combinations listed in Supplemental Table 2 were sown in June 2023 at the Hokkaido Agricultural Research Center (42°88.3ʹN, 143°05.5ʹE). Each cross was performed in a mesh-netted plot of two rows 1 m long and 60 cm between rows, with 40 seeds were sown per row for the SI line and 20 seeds per row for the SC line. For the development of parent lines, 40 SI plants including pin and thrum and 20 SC plants were grown. For the production of F_1_ seeds, 10 to 20 plants of SI plants with uniform flower type, pin or thrum were selected. For SI × SI crosses, pin plants of one line were grown in one row and thrum plants from the other line were grown in the other row. For SI × SC crosses, pin plants of the SI line were planted in one row and plants of the SC line were planted in the other row (Matsui and Yasui 2020). Plants were pollinated by flies. Seeds were harvested at maturity, dried, and stored in a refrigerator.

### Production of F_1_ seeds in a glasshouse with hand-pollination for RNA sequencing

To clarify whether genes related to rutin synthesis derived from pollen parents are expressed in F_1_ seeds, we crossed HR8 × PL4 and HR8 × HR8. The plants were grown in pots in a glasshouse at the Institute of Crop Science (36°03.0ʹN, 140°09.9ʹE). As HR8 is SI, each plant had a different genotype. To detect alleles from the pollen parent, we prepared two HR8 pin plants (HR8-pin-A and HR8-pin-B) as maternal plants; and two HR8 thrum plants (HR8-thrum-C and HR8-thrum-D) and two PL4 long-homostyle plants (PL4-LH-A and PL4-LH-B) as pollen parents. Using different branches, we crossed HR8-pin-A × PL4-LH-A, HR8-pin-A × HR8-thrum-C, HR8-B × HR8-thrum-D, and HR8-B × PL4-LH-B (Supplemental Fig. 1). Each cross was hand pollinated and then bagged to prevent crosses with other plants. Immature seeds were harvested 10–20 days after crossing and frozen quickly in liquid nitrogen. Four seeds derived from each cross combination were used for RNA extraction.

### Measurement of rutin contents by high-performance liquid chromatography (HPLC)

One dehulled seed was placed in a 2.0-mL tube with a small bead and crushed (2500 rpm for 60 s) in a Micro Smash MS-100 cell disruptor (Tomy Seiko, Tokyo, Japan). Then 1 mL of 80% ethanol was added, and the samples were incubated at 37°C for 3 h. They were centrifuged at 21 000 × *g* for 5 min at 4°C and the supernatant was analyzed for rutin content by HPLC (Ishiguro *et al.* 2016). The HPLC system consisted of two pumps (LC-20AD), an autoinjector (SIL-20AC), and a column oven (CTO-20AC, all from Shimadzu, Kyoto, Japan). Into a reversed-phase column (3 μm, 150 mm × 2 mm i.d., Cadenza CD-C18, Imtakt Co., Ltd., Kyoto, Japan) at 40°C was injected 2 μL of extract. The mobile phase was composed of phase A (7.5% v/v acetonitrile containing 0.1% v/v trifluoroacetic acid [TFA]) and phase B (50% v/v acetonitrile containing 0.1% v/v TFA). Samples were eluted with a 35% solution of phase B in phase A at 0.3 mL/min for 18 min. Rutin was identified from the retention time and UV-vis spectra of a standard, and was quantified against an external standard on a calibration curve based on detection at 360 nm.

### Maternal and pollen effects on rutin content

Five plants were selected from each row (Supplemental Table 2). The rutin content of each of 15 seeds per plant was measured. Means were compared by Fisher’s Least Significant Difference (LSD) test at *P* = 0.05, 0.01, or 0.001 in BellCurve for Excel software (Social Survey Research Information Co., Ltd., Tokyo, Japan).

Maternal and pollen effects on the rutin content were calculated based on the method of Wang *et al.* (2010). The rutin content of F_1_ seeds, *F_1_*, was calculated as:


$$F_{1}={mP}_{\text{m}}+(1-m)P_{\text{p}}$$

where *P*_m_ and *P*_p_ are the rutin contents of the maternal and pollen parents, and *m* is the maternal effect, calculated as *m* = (*F_1_* – *P*_p_)/(*P*_m_ – *P*_p_), and therefore the pollen parent effect = 1 – *m*. The mid-parental value (MP) = (*P*_m_ + *P*_p_)/2.

### RNA sequencing analysis for identifying alleles derived from pollen parent and calculation of the ratio of alleles derived from pollen parent

We investigated the expression of genes related to rutin synthesis in the phenylpropanoid and flavonoid biosynthesis pathways in maturing seeds of HR8 pin plants × PL4 pollen parent and of HR8 pin plants × HR8 thrum plants to confirm that any allelic differences are not caused by natural variation in HR8.

Maturing seeds were frozen in liquid nitrogen and homogenized with a mortar and pestle. Total RNA was extracted from the seeds with a Maxwell RSC Plant RNA Kit in a Maxwell RSC Instrument (Promega) according to the manufacturer’s protocol. A Next Generation Sequencing library was constructed with a TruSeq stranded mRNA Library Preparation Kit (Illumina), and RNAs were sequenced on a NovaSeq 6000 sequencer to generate 150-bp paired-end reads. Short reads were cleaned in fastp software (v0.23.4) (Chen *et al.* 2018), and the first and last 6 bases of each locus were trimmed because the reliability of the ratio of nucleotide in each SNP was low due to low quality of base-call in this region. The cleaned short reads were mapped to the cDNA sequence of PL4 (Fawcett *et al.* 2023) in Bowtie 2 software (v2.5.1) with default parameters (Langmead and Salzberg 2012). The mapped data were visualized in Integrative Genomics Viewer (IGV) software (Robinson *et al.* 2011). Additionally, transcripts per million (TPM) values were calculated using RSEM software (v1.3.3) (Li and Dewey 2011).

Genes encoding enzymes involved in rutin synthesis were selected based on the report by Fawcett *et al.* (2023). After the RNA-seq data were mapped to the reference sequence, loci of expressed genes with TPM >1.0 were determined and SNPs were searched in IGV. Alleles derived from pollen parents were detected by comparison of the results between HR8 × HR8 and HR8 × PL4.

We also estimated the ratio of expression of alleles derived from pollen parent by calculating the ratio of nucleotides derived from pollen parent on each SNP indicated by IGV to determine the relationship between the ratio of expression of alleles derived from pollen parent and rutin content in seed (Supplemental Fig. 2).

### Accession numbers

The raw RNA-seq data were submitted to the DDBJ Sequence Read Archive (https://www.ddbj.nig.ac.jp/dra/index-e.html) under accession numbers DRA018730, DRA018757, DRA018758, and DRA018772.

## Results

### Rutin contents among materials

The average rutin contents of KTM, KTW, RRN and KTY were all 0.15–0.21 mg/g, and that of HR8 was 0.6 mg/g. Because these are all SI and thus heterozygosity is high, SD had a wide range (0.12–0.41; Table 1). On the other hand, the average rutin contents of SC lines PL4 and LoR were both 0.01–0.06 mg/g, with stable SDs (0.02–0.03; Table 1). From these results, we classified these lines into three groups by rutin content—low (<0.1 mg/g), medium (0.1–0.5 mg/g), and high (>0.5 mg/g)—and investigated the effects of the pollen parent with crosses among these groups (Supplemental Table 1).

### Effect of pollen parent on rutin content

**Fig. 2.**
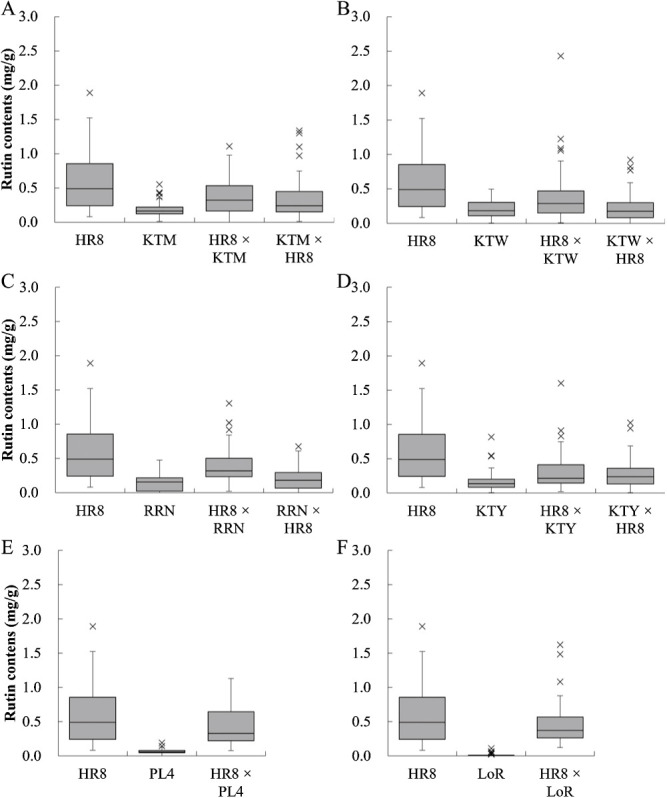
Distribution of rutin contents of F_1_ seeds and their parent lines. HR8 was crossed with (A) KTM, (B) KTW, (C) RRN, (D) KTY, (E) PL4, (F) LoR. The rutin content was measured for each of the 15 seeds from five plants of each lines. The box plots represent the median, interquartile range (IQR), and whiskers extending to 1.5 × IQR. In crosses with HR8 as the maternal parent and (A) KTM, (B) KTW, (C) RRN, or (D) KTY as the pollen parents, seed rutin content was significantly lower than that of HR8. Conversely, when HR8 was the pollen parent, seed rutin content was significantly higher or marginally higher depending on the maternal parent ((A) KTM, (B) KTW, (C) RRN, or (D) KTY). In crosses with (E) PL4 or (F) LoR, seed rutin content was significantly lower than that of HR8. Statistical analyses were performed using LSD test. Significant differences are summarized in Table 2.

Because only HR8 had a high rutin content, we used this line in all crossing combinations. When HR8 as the maternal parent was crossed with KTM, KTW, RRN and KTY as the pollen parents, the rutin contents of the seeds (0.31–0.40 mg/g; Table 2) were significantly lower than that of HR8 (0.60 mg/g; Table 1), indicating that the rutin content was influenced by the pollen parent (Fig. 2A–2D).

Similarly, when KTM and KTY as the maternal parents were crossed with HR8 as the pollen parent, the rutin contents of the seeds (0.32, 0.28 mg/g; Table 2) were significantly higher than that of each maternal parent (0.18, 0.17 mg/g; Fig. 2A, 2D, Table 1). On the other hand, when KTW and RRN as the maternal parents were crossed with HR8 as the pollen parent, the rutin content of the seeds (0.22, 0.21 mg/g; Table 2) was only marginally higher than that of the seed parents (0.21, 0.15 mg/g; Fig. 2B, 2C, Table 1).

In the crosses between HR8 and PL4 or LoR, the rutin contents of the seeds (0.44 and 0.45 mg/g; Table 2) were significantly lower than those of HR8 (0.60 mg/g; Table 1) and higher than those of PL4 or LoR (0.06, 0.01 mg/g; Fig. 2E, 2F, Table 1), indicating that the rutin content was influenced by the pollen parent.

### Identification of expressed genes derived from pollen parents

**Fig. 3.**
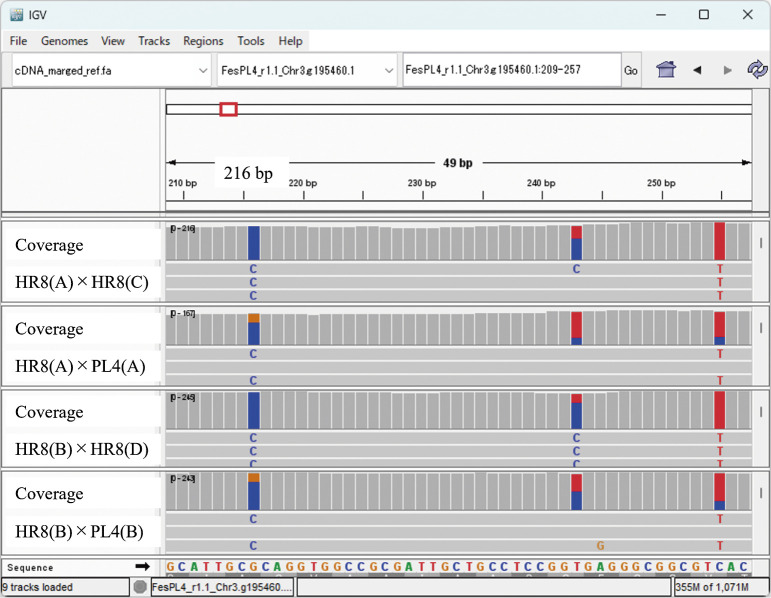
Example of gene expression in maturing seeds of (rows 1 and 3) HR8 × HR8 and (rows 2 and 4) HR8 × PL4. The reference sequence is PL4, and nucleotides that are the same as in PL4 are shown in gray. Proportions of nucleotides that differ from those in PL4 are shown by color, and the nucleotide is indicated. For example, at 216 bp, the nucleotide in rows 1 and 3 (HR8 × HR8) is C (blue), whereas that in rows 2 and 4 (HR8 × PL4) are C (blue) and G (orange). This difference indicates that the “G” allele of the pollen parent “PL4” was expressed.

Twenty-two genes encoding enzymes in the rutin biosynthesis pathway—approximately 35% of involved genes—were expressed with TPM >1.0 in maturing seeds (Table 3, Supplemental Table 3), indicating the synthesis of rutin during seed development. Allele sequences derived from the pollen parent PL4 were recognized at 14 loci (Fig. 3, Table 3). Furthermore, sequences that cause amino acid variation were identified in 9 loci (Supplemental Table 3).

## Discussion

### Paternal and maternal effects on the rutin content of F_1_ hybrid seeds

Pollen parents have a direct genetic influence on F_1_ seeds in some plants (Denney 1992, Kanade *et al.* 2024). They influence sugar content and enzymes for secondary metabolites in palm fruit (Shahsavar and Shahhosseini 2022) and yield and anthocyanin content in highbush blueberry (Doi *et al.* 2021). However, to our knowledge, there are no reports in buckwheat.

We clarified the effects of the pollen parent on the rutin content of F_1_ seeds through the use of the SC lines PL4 and LoR and of the high-rutin-content line HR8. As most buckwheat cultivars are SI, it is difficult to develop lines in which rutin content is fixed, especially at a high level, because of the absence of genetic information. Using only common cultivars, it would be difficult to determine whether rutin content is influenced by the pollen parent or simply varies within a line. The low rutin contents of PL4 and LoR are fixed. So when they were crossed with HR8, the effect of the pollen parent was clearly detected (Fig. 2E, 2F).

HR8 was developed by long-term recurrent selection for high rutin content and has a significantly higher content than the other SI lines, although variation is still large. Significant differences in the rutin content between the F_1_ seeds and the maternal parent lines were found when HR8 as the maternal parent was crossed with the other SI lines as the pollen parents and when KTM and KTY as the maternal parents were crossed with HR8, but not when KTW and RRN as the maternal parents were crossed with HR8 as the pollen parent. The lack of significant differences in the rutin content between F_1_ seeds and the maternal parents in some cross combination would be probably caused by the broad range of both lines. HR8 can help clarify the effect of the pollen parent on the rutin content of seeds, but the development of better-fixed lines would be more useful.

In crosses between medium-rutin-content lines KTM, KTW, RRN and KTY as the maternal parents and HR8 as the pollen parent, the effect value of the pollen parent on rutin content averaged 0.19 (range, 0.02–0.35; Table 2), lower than that of the opposite crosses including crosses between HR8 and PL4 or LoR (0.46; range, 0.26–0.68). Although we don’t have clear answer about this phenomenon, one of the reasons seems the broad distribution of rutin content in HR8 (Fig. 2, Table 1). When HR8 with low rutin content is used as the pollen parent, its effect is likely to be smaller. In contrast, when HR8 with low rutin content is used as the seed parent, the effect of the pollen parent from mid- and low-rutin-content line becomes more pronounced. For the accurate assess of the degree of the pollen parent effect, a SC high-rutin line with stable rutin content would be needed.

### Detection of alleles derived from pollen parents and relationship between the ratio of alleles and rutin contents

If rutin contents are influenced by pollen parents, alleles related to rutin synthesis of the pollen parent should be expressed during seed development. We detected the expression of pollen parent alleles in F_1_ seeds for several genes encoding enzymes involved in rutin synthesis. Approximately 60% of the alleles derived from the pollen parents were recognized in the expressed loci (Table 3). Approximately 40% of the loci, the ratio of pollen alleles was 20%–40% (Table 4), comparable to the influence of the pollen parent on the rutin content of PL4 × HR8 (0.29; Table 2). However, some genes lay outside this range, in which the lowest influence of pollen parent on genes was 9.6% and the highest was 74.2% (Table 4). Buckwheat seeds are composed of a diploid embryo, a triploid endosperm, and a diploid testa derived from the maternal parent, with different rutin contents, which are highest in the embryo (Suzuki *et al.* 2002). The expression levels of genes involved in rutin synthesis seem to depend on maturity stages (Penin *et al.* 2021). Thus, a larger embryo size and a larger proportion of embryo in the seed would mean a higher rutin content. The expression of genes related to rutin synthesis may increase as the embryo grows larger, but the embryo’s size in the seed may be regulated by a number of genes. Further study will be needed to clarify the relationships between the ratio of each allele and rutin content. Examining the rutin content at each maturation stage and determining the ratio of pollen parent allele using qPCR could provide a clearer understanding of their relationship with rutin content.

### Inference of genes affecting rutin content

Among the nine genes with sequences confirmed to have amino acid variation (Supplemental Table 3), the PAL gene (FesPL4_r1.1_Chr8.g155630.1) and the F3′H gene (FesPL4_r1.1_Chr8.g248260.1) exhibited a high ratio of pollen parent alleles (Table 4), suggesting that these genes may influence rutin content.

In particular, activation of the F3′H gene contributes to increased rutin content in *Fagopyrum tataricum* (Li *et al.* 2022). On the other hand, F3′H is an enzyme involved not only in rutin synthesis but also in anthocyanin synthesis. According to Zhang *et al.* (2019), F3′H increases anthocyanin content while decreasing rutin content. Furthermore, multiple isozymes of F3′H exist, each of which may function differently depending on the substrate. Whether FesPL4_r1.1_Chr8.g248260.1, identified in this study, contributes directly to increasing rutin content based on the HR8-derived sequence or functions to suppress the decrease in rutin content remains unclear at this study. However, it is highly likely that FesPL4_r1.1_Chr8.g248260.1 affects rutin content. Future research focusing on this gene is expected to contribute to the development of high-rutin cultivars.

## Author Contribution Statement

SO and K. Matsui conceived and designed the experiments. SO and K. Matsui crossed the plants. SO, TH, and KI measured rutin by HPLC. TH and K. Matsushima developed plant materials. SO, YY, and K. Matsui performed RNA sequencing analysis. SO and K. Matsui wrote the manuscript. All authors edited and approved the final manuscript.

## Supplementary Material

Supplemental Figures

Supplemental Tables

## Figures and Tables

**Table 1.** Parental lines and seed rutin contents

Parent	Average rutin content (mg/g)	SD
HR8	0.60	0.41
PL4	0.06	0.03
LoR	0.01	0.02
KTM	0.18	0.09
KTW	0.21	0.13
RRN	0.15	0.12
KTY	0.17	0.14

Flower type and self-compatibility of these lines are shown in Supplemental Table 2.

**Table 2.** Parental effects on the rutin contents of F_1_ seeds

Cross combination by rutin contents	Cross combination of lines	Cross No.*^a^*	Mid-parental value	Rutin content (mg/g)	SD	Significance *^b^*		Maternal effect value	Pollen parent effect value
						P_m_ vs. F_1_	P_p_ vs. F_1_		
High × medium or low	HR8 × KTM	a1	0.39	0.38	0.26	***	***	0.49	0.51
	HR8 × KTW	b1	0.40	0.36	0.36	***	**	0.40	0.60
	HR8 × RRN	c1	0.37	0.40	0.26	***	***	0.56	0.44
	HR8 × KTY	d1	0.38	0.31	0.26	***	**	0.32	0.68
	HR8 × PL4	e	0.33	0.44	0.28	**	***	0.71	0.29
	HR8 × LoR	f	0.30	0.45	0.27	**	***	0.74	0.26
	average	–	–	–	–	–	–	0.54	0.46
Medium × high	KTM × HR8	a2	0.39	0.32	0.28	**	***	0.65	0.35
	KTW × HR8	b2	0.40	0.22	0.19	0.87	***	0.98	0.02
	RRN × HR8	c2	0.37	0.21	0.16	0.16	***	0.86	0.14
	KTY × HR8	d2	0.38	0.28	0.20	*	***	0.75	0.25
	average	–	–	–	–	–	–	0.81	0.19

Flower type and self-compatibility of these lines are shown in Supplemental Table 2. *^a^* Cross No. with the same letter indicates that they were grown and crossed in the same plot. *^b^* Means were compared by LSD test. P_m_, maternal parent; P_p_, pollen parent. Significant differences at *0.05, **0.01, and ***0.001 probability levels.

**Table 3.** Gene families encoding enzymes involved in the rutin biosynthesis pathway and the number of genes expressed during seed formation

Enzyme	Number of loci		
	Detected in reference	Expressed	Pollen parent alleles detected
Phenylalanine ammonia-lyase (PAL)	4	4	3
Cinnamate-4-hydroxylase (C4H)	6	2	1
4-Coumarate CoA ligase (4CL)	2	2	1
Chalcone synthase (CHS)	13	5	3
Chalcone isomerase (CHI)	3	1	1
Flavanone-3-hydroxylase (F3H)	2	2	1
Flavonoid-3ʹ-hydroxylase (F3ʹH)	2	2	1
Flavonoid-3ʹ-5ʹ-hydroxylase (F3ʹ5ʹH)	16	3	3
Flavonol synthase (FLS)	2	1	0
Glycosyltransferase (GTR)*^a^*	12	(1)	(1)
Total *^a^*	62	22	14

*^a^* Gene for GTR was expressed in only one plant. The total does not include GTR.

**Table 4.** SNPs detected in expressed genes related to flavonoid biosynthesis and the ratio of alleles derived from pollen parent

Enzyme	Locus	Total number of nucleotides	HR8-pin-A × PL4-LH-A						HR8-pin-B × PL4-LH-B				
			Number of SNPs*^a^*			Ratio of pollen parent allele*^b^*			Number of SNPs*^a^*			Ratio of pollen parent allele*^b^*	
			Pattern I	Pattern II		Average ± SE			Pattern I	Pattern II		Average ± SE	
PAL	FesPL4_r1.1_Chr3.g195460.1	2173	22	3		30.3	1.94		15	10		16.9	4.02
	FesPL4_r1.1_Chr4.g269240.1	2110	20	18		22.5	9.80		43	5		14.6	5.60
	FesPL4_r1.1_Chr8.g155630.1	2113	4	12		44.4	6.63		8	7		47.1	6.55
C4H	FesPL4_sc0109.1.g001280.1	1516	39	5		20.7	2.05		22	7		26.7	5.11
4CL	FesPL4_r1.1_Chr4.g271010.1	1642	13	1		38.5	–		35	2		18.0	2.00
CHS	FesPL4_r1.1_Chr4.g217000.1	1183	3	7		68.0	3.15		3	7		66.1	5.61
	FesPL4_r1.1_Chr7.g094080.1	1180	5	0		–	–		4	1		74.2	–
	FesPL4_r1.1_Chr7.g094660.1	1180	5	0		–	–		4	1		9.6	–
CHI	FesPL4_r1.1_Chr3.g000530.1	772	11	0		–	–		10	0		–	–
F3H	FesPL4_r1.1_Chr5.g258370.1	1105	10	3		21.4	0.91		13	3		20.0	2.52
F3ʹH	FesPL4_r1.1_Chr8.g248260.1	1588	9	16		54.9	8.55		8	16		53.9	7.66
F3ʹ5ʹH	FesPL4_r1.1_Chr4.g265140.1	1834	13	1		36.4	–		15	0		–	–
	FesPL4_r1.1_Chr4.g265180.1	1876	31	1		11.8	–		18	0		–	–
	FesPL4_r1.1_Chr4.g265190.1	1576	5	0		–	–		5	0		–	–

*^a^* Two different SNP patterns are recognized because HR8 is SI and keeps heterozygosity in many loci (Supplemental Fig. 2). *^b^* -: The SNPs in pattern I shown in Supplemental Fig. 2 were detected. However, the SNPs in pattern II were not detected, therefore the ratio of pollen parent allele could not be calculated.
