# Supplementary material for: Pollen parent affects rutin content of seeds of buckwheat (Fagopyrum esculentum)
Source: Breed Sci. 2025 Jun 18;75(3):179–86. doi: 10.1270/jsbbs.24085 (PMC12457789; doi:10.1270/jsbbs.24085)
Supplement: Supplementary file 1 — Supplemental Figures [file 75_179_s1.pdf]

doi: 10.1270/jsbbs.24085

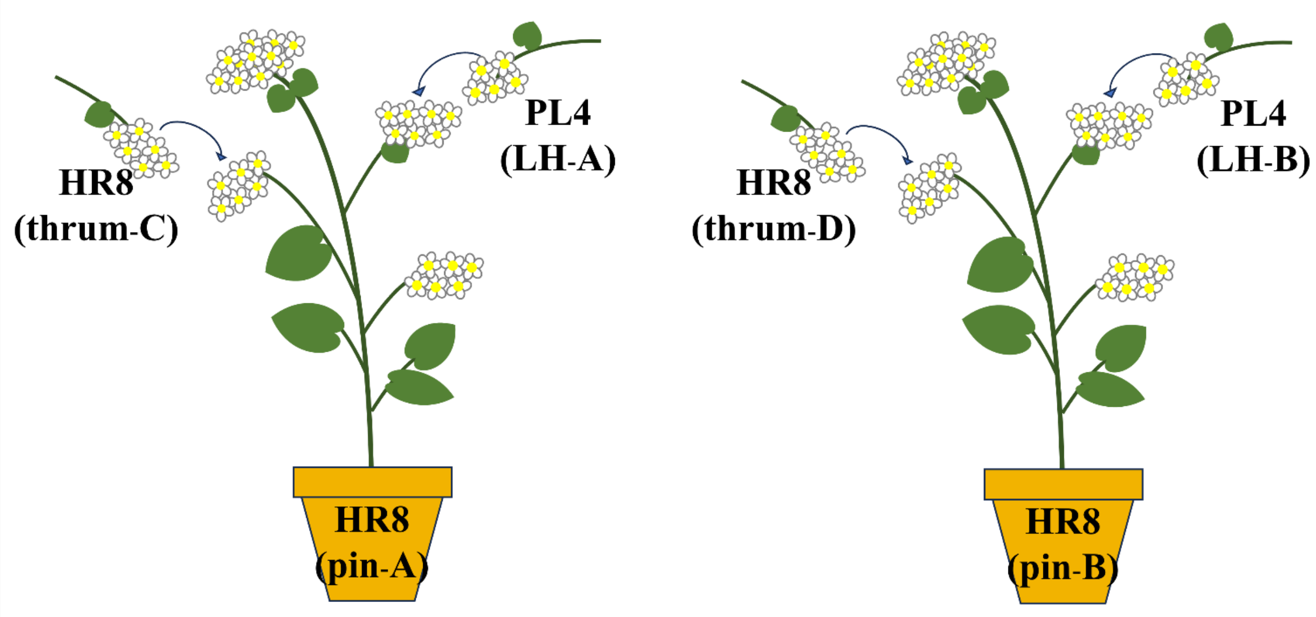

Supplemental Fig. 1. Crossing of HR8 with HR8 and PL4.
Four different HR8 plants (HR8-pin-A, HR8-pinB, HR8-thrum-C, HR8-thrum-D) and two different PL4 plants (PL4-LH-A, PL4-LH-B) were used for crossing. HR8-pin × PL4-LH and HR8-pin × HR8-thrum crosses were performed on different branches of HR8-pin.

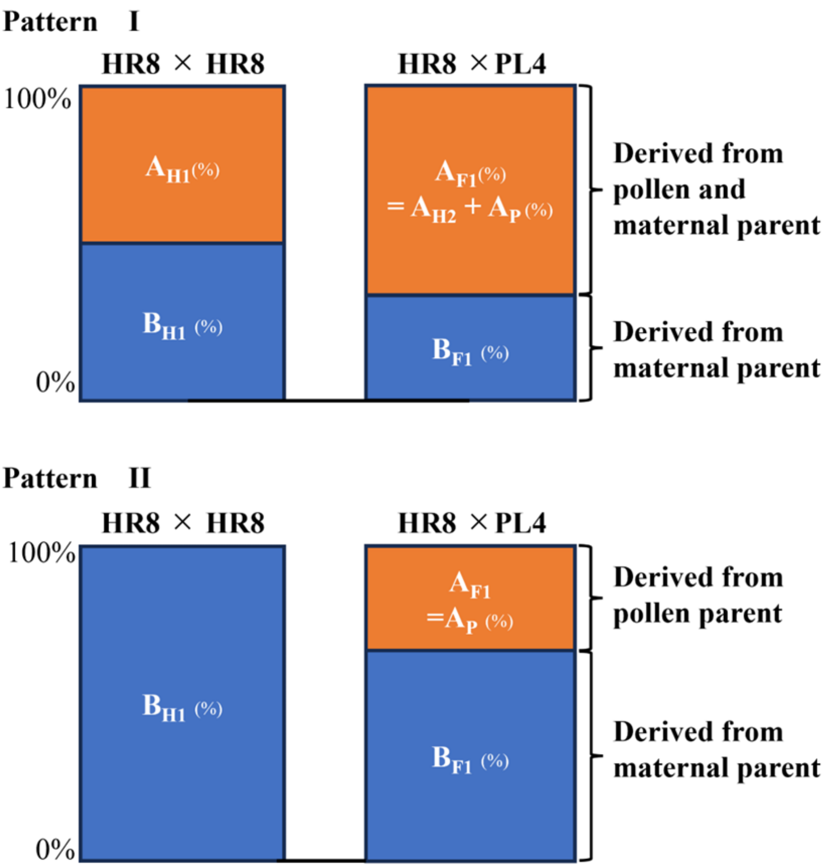

Supplemental Fig. 2. Calculation of the ratio of nucleotide which was derived from pollen parents on each SNP.

To estimate the ratio of pollen-derived alleles, the following method based on the ratio of nucleotide on each SNP was used. As PL4, the pollen parent, is SC, there is only one nucleotide at any SNP. But because HR8 is SI, two nucleotides could appear at a SNP. Therefore, SNPs can show two patterns of polymorphism: polymorphism in both cross combinations, when HR8 has two nucleotides at a SNP due to heterozygosity (Pattern I); and polymorphism in HR8 × PL4 and no polymorphism in HR8 × HR8 (Pattern II).

Here, a nucleotide that is the same as in PL4 is called nucleotide “A”, and one that is different from that in PL4 is called nucleotide “B”. The ratio of nucleotides A and B in HR8 × HR8 were determined by IGV, and are shown here as A_{H1} and B_{H1}. The ratio of nucleotides A and B in HR8 × PL4 were also determined by IGV, and are shown here as A_{F1} and B_{F1}. A_{F1} represents the combined values of the maternal parent HR8 (A_{H2}) and of the pollen parent PL4 (A_P): $A_{F1} = A_{H2} + A_P$.

In the case of Pattern I, determining A_P on each SNP is difficult. At the SNP, when the HR8 has nucleotides A and B, A_{F1} represents the combined values of A_{H2} and A_P ($A_{F1} = A_{H2} + A_P$), thus A_P is not clearly shown. On the other hand, in the case of Pattern II, A_P on each SNP can be determined. Since HR8 does not have nucleotide A, the value of A_{F1} indicates only A_P ($A_{F1} = A_P$).

Therefore, in this study, A_P at each SNP was examined only Pattern II. Finally, the value of A_P in each SNP was averaged for each gene locus to estimate the ratio of allele derived from the pollen parent (Table 4).
