# Supplementary material for: Pollen parent affects rutin content of seeds of buckwheat (Fagopyrum esculentum)
Source: Breed Sci. 2025 Jun 18;75(3):179–86. doi: 10.1270/jsbbs.24085 (PMC12457789; doi:10.1270/jsbbs.24085)
Supplement: Supplementary file 2 — Supplemental Tables [file 75_179_s2.pdf]

doi: 10.1270/jsbbs.24085

Supplemental Table 1. Parental lines used.

Cultivar/Line	Abbreviation	Rutin content[a]	Self-compatible (SC) or self-incompatible (SI)	Harvest date
Kitamitsuki	KTM	medium	SI	16-Oct
Kitawasesoba	KTW	medium	SI	16-Oct
Reranokaori	RRN	medium	SI	17-Oct
Kitayuki	KTY	medium	SI	18-Oct
High Rutin content line No. 8	HR8	high	SI	21-Oct
Kyushu PL4	PL4	low	SC	13-Oct
Low Ruin content line	LoR	low	SC	13-Oct

The sowing date is June 8th.

[a] Rutin contents of each line were roughly classified in order to design cross combinations.

Supplemental Table 2. Cross combinations and flower types and harvest date

Cross combination	Cross No.[a]	Parental line		Harvest date
		Maternal	Paternal	
SI × SI [b]	a1	HR8 (pin)	KTM (thrum)	17-Oct
	a2	KTM (thrum)	HR8 (pin)	17-Oct
	b1	HR8 (pin)	KTW (thrum)	17-Oct
	b2	KTW (thrum)	HR8 (pin)	17-Oct
	c1	HR8 (pin)	RRN (thrum)	18-Oct
	c2	RRN (thrum)	HR8 (pin)	18-Oct
	d1	HR8 (pin)	KTY (thrum)	19-Oct
	d2	KTY (thrum)	HR8 (pin)	19-Oct
SI × SC	e	HR8 (pin)	PL4 (homo)	3-Oct
	f	HR8 (pin)	Low (homo)	6-Oct

The sowing date is June 8th.

[a] Cross No. with the same letter indicates that they were grown and crossed in the same plot.

[b] SI, self-incompatible; SC, self-compatible.

Supplemental Table3. TPM of rutin biosynthesis genes, SNP counts, and amino acid variation positions in HR8×HR8 and HR8×PL4

Enzyme	Locus	TPM						Number of SNPs		Nucleotide position causing amino acid variation[a]
		HR8-pin-A ×HR8-thrum-C	HR8-pin-B ×HR8-thrum-D	Average of HR8×HR8	HR8-pin-A ×PL4-LH-A	HR8-pin-B ×PL4-LH-B	Average of HR8×PL4	HR8-pin-A ×PL4-LH-A	HR8-pin-B ×PL4-LH-B	
PAL	FesPL4_r1.1_Chr3.g195460.1	17.16	17.99	17.58	14.45	19.08	16.77	25	25	-
	FesPL4_r1.1_Chr4.g269240.1	7.25	7.62	7.44	6.52	7.24	6.88	38	48	(640), (649)
	FesPL4_r1.1_Chr7.g002240.1	13.52	3.22	8.37	14.22	8.70	11.46	0	0	-
	FesPL4_r1.1_Chr8.g155630.1	17.19	15.77	16.48	20.13	17.12	18.63	16	15	221, 577, 647, 692, 1808
C4H	FesPL4_r1.1_Chr1.g274200.1	0.05	0.03	0.04	0.15	0.10	0.13	-	-	-
	FesPL4_r1.1_Chr8.g236380.1	0.19	0.07	0.13	0.50	0.29	0.40	-	-	-
	FesPL4_r1.1_Chr8.g236440.1	0.24	0.16	0.20	0.26	0.16	0.21	-	-	-
	FesPL4_r1.1_Chr8.g236500.1	19.61	16.57	18.09	25.74	22.36	24.05	0	0	-
	FesPL4_r1.1_Chr8.g236520.1	0.00	0.00	0.00	0.00	0.00	0.00	-	-	-
	FesPL4_sc0109.1.g001280.1	36.21	20.75	28.48	45.79	33.70	39.75	44	29	182, 1030-1031, 1276
4CL	FesPL4_r1.1_Chr3.g221480.1	7.55	3.28	5.42	5.86	4.17	5.02	0	0	-
	FesPL4_r1.1_Chr4.g271010.1	15.54	10.52	13.03	15.89	11.43	13.66	14	37	439, 443
CHS	FesPL4_r1.1_Chr2.g208620.1	0.00	0.00	0.00	0.00	0.00	0.00	-	-	-
	FesPL4_r1.1_Chr3.g006440.1	0.00	0.00	0.00	0.00	0.00	0.00	-	-	-
	FesPL4_r1.1_Chr3.g203560.1	42.25	20.25	31.25	27.52	23.07	25.30	0	0	-
	FesPL4_r1.1_Chr3.g250660.1	0.00	0.00	0.00	0.03	0.00	0.02	-	-	-
	FesPL4_r1.1_Chr3.g250940.1	0.00	0.00	0.00	0.00	0.00	0.00	-	-	-
	FesPL4_r1.1_Chr4.g217000.1	15.63	16.88	16.26	43.99	28.51	36.25	10	10	-
	FesPL4_r1.1_Chr4.g259930.1	0.65	0.04	0.35	1.45	0.62	1.04	-	-	-
	FesPL4_r1.1_Chr4.g260090.1	0.51	0.00	0.26	0.88	0.29	0.59	-	-	-
	FesPL4_r1.1_Chr4.g261060.1	10.65	1.79	6.22	37.85	16.21	27.03	0	0	-
	FesPL4_r1.1_Chr7.g037250.1	0.00	0.00	0.00	0.00	0.00	0.00	-	-	-
	FesPL4_r1.1_Chr7.g094080.1	39.03	18.04	28.54	38.62	27.05	32.84	5	5	-
	FesPL4_r1.1_Chr7.g094660.1	33.51	15.69	24.60	28.22	27.68	27.95	5	5	-
CHI	FesPL4_r1.1_Chr3.g000530.1	12.83	8.26	10.55	12.23	8.37	10.30	11	10	-
	FesPL4_r1.1_Chr3.g000540.1	0.07	0.17	0.12	0.00	0.00	0.00	-	-	-
	FesPL4_sc0330.1.g000190.1	0.00	0.00	0.00	0.00	0.00	0.00	-	-	-
F3H	FesPL4_r1.1_Chr5.g258370.1	12.04	9.92	10.98	13.04	8.84	10.94	13	16	(463), (472)
	FesPL4_r1.1_Chr8.g147040.1	10.41	4.35	7.38	14.00	6.42	10.21	0	0	-
F3'H	FesPL4_r1.1_Chr8.g176630.1	11.15	11.96	11.56	9.96	12.01	10.99	0	0	-
	FesPL4_r1.1_Chr8.g248260.1	16.06	8.27	12.17	18.32	12.35	15.34	25	24	97, (226), 917

[a] ( ) indicates tha nucleotide position of the SNP detected only in one of two plants.

Supplemental Table3. TPM of rutin biosynthesis genes, SNP counts, and amino acid variation positions in HR8×HR8 and HR8×PL4 (continued)

Enzyme	Locus	TPM						Number of SNPs		Nucleotide position causing amino acid variation[a]
		HR8-pin-A ×HR8-thrum-C	HR8-pin-B ×HR8-thrum-D	Average of HR8×HR8	HR8-pin-A ×PL4-LH-A	HR8-pin-B ×PL4-LH-B	Average of HR8×PL4	HR8-pin-A ×PL4-LH-A	HR8-pin-B ×PL4-LH-B	
F3'5'H	FesPL4_r1.1_Chr4.g265000.1	0.12	0.13	0.13	0.17	0.11	0.14	-	-	-
	FesPL4_r1.1_Chr4.g265010.1	0.00	0.00	0.00	0.29	0.00	0.15	-	-	-
	FesPL4_r1.1_Chr4.g265020.1	0.02	0.02	0.02	0.04	0.14	0.09	-	-	-
	FesPL4_r1.1_Chr4.g265040.1	0.00	0.00	0.00	0.18	0.10	0.14	-	-	-
	FesPL4_r1.1_Chr4.g265050.1	0.13	0.00	0.07	0.06	0.06	0.06	-	-	-
	FesPL4_r1.1_Chr4.g265060.1	0.32	0.26	0.29	0.29	0.22	0.26	-	-	-
	FesPL4_r1.1_Chr4.g265070.1	0.21	0.72	0.47	0.20	0.77	0.49	-	-	-
	FesPL4_r1.1_Chr4.g265110.1	0.00	0.00	0.00	0.00	0.00	0.00	-	-	-
	FesPL4_r1.1_Chr4.g265120.1	0.06	0.00	0.03	0.00	0.00	0.00	-	-	-
	FesPL4_r1.1_Chr4.g265130.1	0.00	0.00	0.00	0.00	0.00	0.00	-	-	-
	FesPL4_r1.1_Chr4.g265140.1	3.71	7.18	5.45	3.39	2.82	3.11	14	15	412, 604, 746, 749, 765, 780, (785), 822, 984, 1003, 1048, 1373
	FesPL4_r1.1_Chr4.g265170.1	0.06	0.27	0.17	0.03	0.33	0.18	-	-	-
	FesPL4_r1.1_Chr4.g265180.1[b]	0.81	1.64	1.23	3.31	1.49	2.40	32	18	854, 1330, 1366, 1369,1385
	FesPL4_r1.1_Chr4.g265190.1	5.22	27.78	16.50	31.67	29.47	30.57	5	5	-
	FesPL4_r1.1_Chr4.g265230.1	0.00	0.00	0.00	0.00	0.00	0.00	-	-	-
	FesPL4_r1.1_Chr5.g241080.1	0.00	0.00	0.00	0.00	0.00	0.00	-	-	-
FLS	FesPL4_r1.1_Chr3.g260020.1	19.42	5.23	12.33	26.96	19.70	23.33	0	0	-
	FesPL4_r1.1_Chr4.g177280.1	0.00	0.00	0.00	0.10	0.00	0.05	-	-	-
GTR	FesPL4_r1.1_Chr2.g006050.1	0.00	0.04	0.02	0.03	0.00	0.02	-	-	-
	FesPL4_r1.1_Chr2.g021740.1	6.96	0.61	3.79	1.75	0.48	1.12	29	-	(521), (574), (673), (1255), (1262), (1295-1296), (1321), (1329), (1337)
	FesPL4_r1.1_Chr6.g202530.1	0.00	0.00	0.00	0.00	0.11	0.06	-	-	-
	FesPL4_r1.1_Chr6.g202930.1	0.00	0.00	0.00	0.00	0.00	0.00	-	-	-
	FesPL4_r1.1_Chr7.g056620.1	0.00	0.00	0.00	0.00	0.00	0.00	-	-	-
	FesPL4_r1.1_Chr7.g056710.1	0.00	0.00	0.00	0.00	0.00	0.00	-	-	-
	FesPL4_r1.1_Chr7.g056740.1	0.00	3.86	1.93	0.00	0.00	0.00	-	-	-
	FesPL4_r1.1_Chr7.g056780.1	0.00	0.00	0.00	0.09	0.10	0.10	-	-	-
	FesPL4_r1.1_Chr7.g056910.1	0.03	0.00	0.02	0.09	0.00	0.05	-	-	-
	FesPL4_r1.1_Chr8.g067190.1	0.00	0.00	0.00	0.00	0.00	0.00	-	-	-
	FesPL4_sc0101.1.g000270.1	0.03	0.00	0.02	0.14	0.03	0.09	-	-	-
	FesPL4_sc0102.1.g000060.1	0.03	0.00	0.02	0.17	0.30	0.24	-	-	-

[a] ( ) indicates tha nucleotide position of the SNP detected only in one of two plants.

[b] The TPM value of FesPL4_r1.1_Chr4.g265180.1 was 0.81 in HR8-pin-A×HR8-thrum-C but exceeded 1 in the other three samples. Therefore, SNP variations were investigated.
